# Chest Tube Drainage Versus Conservative Management as the Initial Treatment of Primary Spontaneous Pneumothorax: A Systematic Review and Meta-Analysis

**DOI:** 10.3390/jcm9113456

**Published:** 2020-10-27

**Authors:** Jong Hyuk Lee, Ryul Kim, Chang Min Park

**Affiliations:** 1 Department of Radiology, Seoul National University Hospital, Seoul National College of Medicine, Seoul 03080, Korea; lee87jh@gmail.com; 2 Department of Internal Medicine, Seoul National University Hospital, Seoul National College of Medicine, Seoul 03080, Korea; chrono0707@kaist.ac.kr

**Keywords:** pneumothorax, conservative treatment, tube drainage, recurrence, complications

## Abstract

**Objectives:** This systematic review and meta-analysis aimed to compare chest tube drainage and conservative management as the initial treatment of primary spontaneous pneumothorax (PSP). **Methods:** Studies including PSP patients who received tube drainage or conservative management as the initial treatment were searched in OVID-MEDLINE and Embase through 14 February 2020. The primary outcome was the relative risk (RR) of PSP recurrence, and secondary outcomes were RRs of PSP resolution and adverse events during treatment. A random-effect model using the Mantel–Haenszel method was used to pool RRs. Subgroup and meta-regression analyses were performed to investigate significant predictors of PSP recurrence. **Results:** In total, 11,922 PSP cases from eight studies were analysed, of which 6344 were treated with tube drainage and 5578 were treated with conservative management. The pooled RR of PSP recurrence for conservative management against tube drainage was 0.98 (95% confidence interval [CI], 0.75–1.28; *p* = 0.894). Subgroup and meta-regression analyses revealed that study design (*p* = 0.816), allocation of the PSP amount in each management group (*p* = 0.191), and assessment time for recurrence had no significant impact on PSP recurrence (*p* = 0.816). There was no publication bias (*p* = 0.475). The risk of adverse events of conservative management was significantly lower than that of tube drainage (pooled RR, 0.22; 95% CI, 0.08–1.15; *p* = 0.003). However, no difference was found between the two groups in terms of PSP resolution (pooled RR, 1.01; 95% CI, 0.9–1.15; *p* = 0.814). **Conclusions:** As the initial treatment for PSP, conservative management is comparable to chest tube drainage in terms of PSP recurrence and resolution after treatment, with fewer adverse events during treatment.

## 1. Introduction

Primary spontaneous pneumothorax (PSP), which refers to spontaneous pneumothorax in otherwise healthy people, is a significant health problem, with a reported annual incidence of 7.4 per 100,000 men and 1.2 per 100,000 women [1,2,3]. PSP is a disease of young people and the recurrence rate of PSP after complete resolution by appropriate management is quite high, with reported rates of 29% within 1 year and 32.1% during the lifetime [3]. Although various parameters, including age, body weight, height, smoking, and bullae or pleural thickening on computed tomography examinations, have been postulated as factors potentially influencing PSP recurrence, there is a lack of consensus on which factors are truly associated with PSP recurrence [3,4,5,6,7,8,9,10].

Recently, a randomized controlled trial (RCT) suggested that recurrence of PSP was more frequent in patients treated with chest tube drainage than in those who received conservative management [11]. Nevertheless, considerable heterogeneity remains in the management for PSP, even among authoritative guidelines [1,2]. For large PSP, the American College of Chest Physicians (ACCP) recommends chest tube drainage to expand the lung [2], while the British Thoracic Society (BTS) suggests that asymptomatic patients may be managed by observation alone, with chest tube drainage reserved for only physiologically unstable patients [1]. A systematic review with a meta-analysis is therefore needed to answer the question of which treatment option—chest tube drainage or conservative management—is better in terms of the recurrence of PSP. Thus, we aimed to conduct a comprehensive review and comparison of chest tube drainage and conservative management as the initial treatment of PSP in terms of recurrence after treatment, resolution, and adverse events during treatment.

## 2. Experimental Section

This systematic review was performed and reported in compliance with the Preferred Reporting Items for Systematic Reviews and Meta-Analyses (PRISMA) guidelines.

### 2.1. Search Strategy

We searched the Embase and OVID-MEDLINE databases to identify relevant publications with the following search terms: (pneumothorax OR pneumothoraces) AND (primary OR spontaneous) AND (intervention* OR conservativ* OR observati*). The initial search was undertaken on 14 February 2020. We included all articles published in English without any limits on publication year.

### 2.2. Eligibility Criteria for Study Selection

We reviewed all the retrieved publications regardless of the study type. The inclusion criteria were as follows: (a) RCTs, prospective or retrospective cohort studies, and case-control studies dealing with PSP, not letters, editorial comments, abstracts, conference materials, case reports, case series, review articles, guidelines, consensus statements, or study protocols; (b) studies with unilateral PSP patients; (c) studies that compared chest tube drainage and conservative management as the initial treatment of PSP; and (d) data described in sufficient detail to extract the final outcomes in terms of PSP recurrence, PSP resolution, and adverse events during the treatment. We excluded studies dealing with secondary spontaneous pneumothorax.

Full-text articles were assessed for eligibility independently by two authors (J.H.L. and R.K.). Any discrepancy was resolved by consensus.

### 2.3. Data Extraction

The following items were extracted from the articles: name of the first author, title of the study, year of publication, study design (prospective vs. retrospective), assessment time for recurrence of PSP, PSP amount in each treatment group, total number of patients, PSP recurrence after treatment, PSP resolution, and adverse events related with the treatment. To avoid errors in the data extraction process, two authors (J.H.L. and R.K.) independently reviewed all eligible articles, extracted the abovementioned data, and compared their results. All data were checked for internal consistency, and disagreements between the two authors were resolved by discussion. In addition to this review process, we tried to contact the corresponding authors of each study via e-mail for missing or unreported data.

### 2.4. Assessment of Risk of Bias

Randomized studies included in this study were assessed using the Cochrane risk of bias tool [12]. Bias was assessed over the following pre-specified domains: random sequence generation, concealment of allocation, blinding, completeness of outcome data, and selective reporting. For assessing the quality of nonrandomized studies, the Newcastle–Ottawa scale was used to evaluate the following three pre-specified domains: selection, comparability, and outcomes [13].

### 2.5. Statistical Analysis

The primary outcome of this study was the recurrence rate of PSP after treatment, defined as the occurrence of ipsilateral pneumothorax after the complete resolution of the previous PSP. Secondary outcomes included the resolution rate of PSP, defined as the rate of patients with complete resolution of PSP, and the rate of adverse events, defined as complications related with the treatment (e.g., severe chest pain, haemothorax, empyema, or subcutaneous emphysema).

Because the study population and the characteristics of the cases of pneumothorax included in this study were considered heterogeneous, a random-effect model using the Mantel–Haenszel method was used to calculating pooled relative risks (RRs), 95% confidence intervals (95% CIs), and *p*-values.

Heterogeneity across the included studies was evaluated using the I^2^ statistic, which was derived from the Cochran Q statistic using the following equation, I^2^ = 100% (Q-df)/Q. An I^2^ statistic >50% was regarded as indicating substantial heterogeneity. Subgroup analysis and meta-regression were performed to investigate the causes of heterogeneity with the following covariates: study design (prospective vs. retrospective), allocation of the PSP amount in each management group (equally allocated study vs. non-equally allocated study), and assessment time for recurrence (within 12 months vs. after 12 months). The potential for publication bias was evaluated with a visual assessment of funnel plots of the standard error against the RR, using the Egger regression test of funnel plot asymmetry [14].

A *p* value of <0.05 was considered to indicate a statistically significant difference. All statistical analyses were performed using R version 4.0.0 (R Project for Statistical Computing, Vienna, Austria).

## 3. Results

### 3.1. Eligible Studies

**Figure 1:**
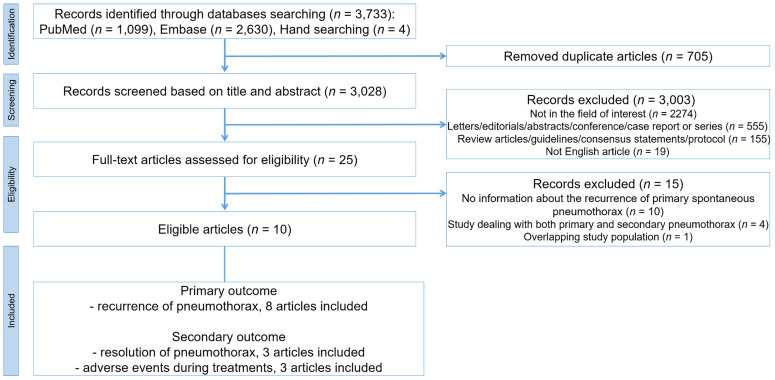
Flow diagram of the literature search.

The search of electronic databases resulted in a total of 3733 studies. After excluding 705 duplicate articles, the abstracts of 3028 studies were examined for relevance to the inclusion criteria and 3003 studies were excluded (study not in the field of interest, *n* = 2274; inappropriate study type, *n* = 710; not written in English, *n* = 19). The remaining 25 studies were thoroughly reviewed in detail and 15 studies were additionally excluded (no information about PSP recurrence, *n* = 10; study dealing with both PSP and secondary spontaneous pneumothorax, *n* = 4; overlapping study population, *n* = 1). Finally, 10 studies were eligible for the analyses of recurrence of PSP after treatment (*n* = 8) [6,9,11,15,16,17,18,19], resolution of PSP (*n* = 3) [11,20,21], and adverse events during treatments (*n* = 3) (Table 1 and Figure 1) [11,16,21].

Regarding the risk of bias, in one RCT, the participants and staff were not blinded to the type of treatment that each participant received because of the open-label study design [11]. All nine nonrandomized studies had a high risk of bias in terms of comparability, and their median score was 6 (range, 4–7).

### 3.2. Recurrence of PSP after Treatment

**Figure 2:**
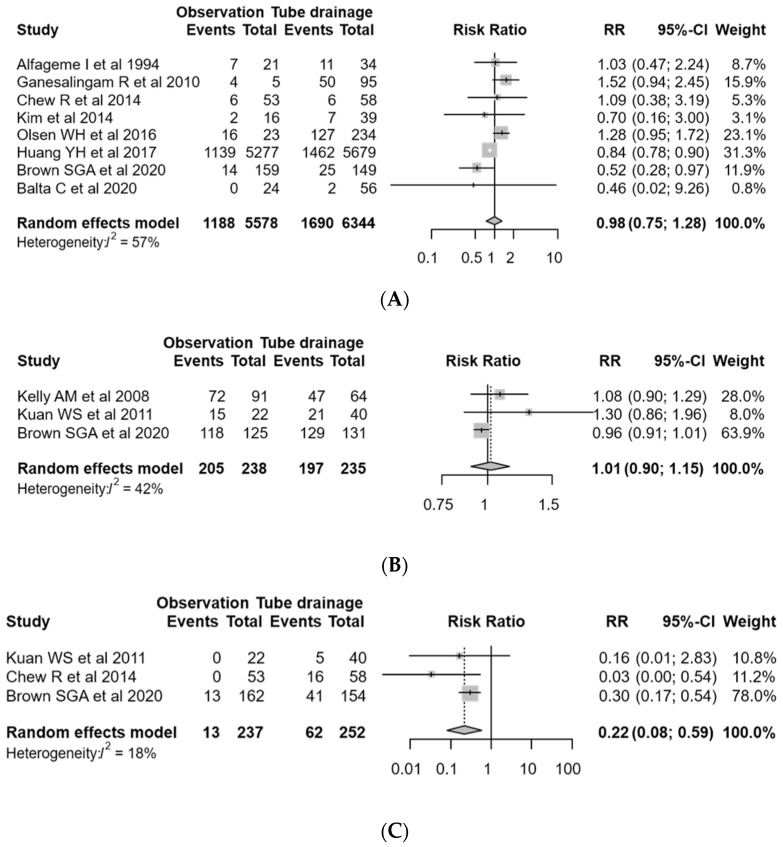
Forest plot comparing conservative management and chest tube drainage for primary spontaneous pneumothorax (PSP). (**A**) Recurrence of PSP, (**B**) resolution of PSP, and (**C**) adverse events during treatment.

Eight studies with 11,922 PSP cases were eligible for assessing the difference in pneumothorax recurrence after treatment [6,9,11,15,16,17,18,19]. Conservative management was provided in 5578 PSP cases, of which 1188 (21.3% of 5578 cases) had recurrence after treatment. In contrast, the other 6344 cases were treated with chest tube drainage, of which 1690 had recurrence (26.6%). The pooled RR of recurrence in the conservative management group relative to the tube drainage group was 0.98 (95% CI, 0.75–1.28; *p* = 0.894; I^2^ = 57%) (Figure 2A). Subgroup and meta-regression analyses revealed that study design (*p* = 0.816), allocation of the PSP amount in each management group (*p* = 0.191), and assessment time for recurrence (*p* = 0.816) did not have a significant impact on pneumothorax recurrence after treatment. (Supplementary Table S1 and Supplementary Figure S1). Specifically, in the subgroup analysis of studies assessing recurrence of PSP within 12 months, the pooled RR for conservative management compared to chest tube drainage was 0.9 (95% CI, 0.75–1.28). There was no publication bias according to the funnel plots (*p* = 0.475; Supplementary Figure S2).

### 3.3. PSP resolution and Treatment-Related Adverse Events

Three studies with 473 PSP cases were eligible for estimating the RR of PSP resolution [11,20,21]. Two hundred thirty-eight cases received conservative management and the remaining 235 cases underwent chest tube drainage as the initial treatment for PSP. Resolution of PSP occurred in 205 (86.1%) and 197 (83.8%) cases in the conservative management group and tube drainage group, respectively. The pooled RR for PSP resolution of conservative management relative to chest tube drainage was 1.01 (95% CI, 0.9–1.15; *p* = 0.814; I^2^ = 42%) (Figure 2B). In the subgroup and meta-regression analyses, study type (*p* = 0.094) and allocation of the PSP amount in each management group (*p* = 0.094) had no significant impact on PSP resolution (Supplementary Table S1 and Supplementary Figure S3).

The analysis of treatment-related adverse events included 489 PSP cases from three studies [11,16,21]. Adverse events during treatment occurred in 13 of the 237 PSP cases receiving conservative management (5.5%) and in 62 of the 252 cases (24.6%) receiving chest tube drainage. The pooled RR of adverse events of conservative management relative to chest tube drainage was 0.22 (95% CI, 0.082–0.591; *p* = 0.003; I^2^ = 18%), demonstrating that the conservative management group had significantly fewer treatment-related adverse events than the chest tube drainage group (Figure 2C). Although there was no heterogeneity in the subgroup analysis according to the study design (I^2^ = 0%), heterogeneity was identified regarding the allocation of the PSP amount in each management group (I^2^ = 57%) (Supplementary Table S1 and Supplementary Figure S4). The most common adverse events were severe chest pain or shortness of breath in both the conservative management group (n=4) and the chest tube drainage group (*n* = 11). Haemothorax and empyema occurred in both the conservative management group (haemothorax, *n* = 3; empyema, *n* = 1) and the tube drainage group (haemothorax, *n* = 10; empyema, *n* = 4). Tension pneumothorax was only reported in the chest tube drainage group (*n* = 3).

## 4. Discussion

Since PSP occurs mainly in young people and recurrence is common, patients with PSP may present to hospitals with recurrent PSP multiple times in their lives [3,22,23]. For this reason, PSP treatment should seek to minimise the recurrence rate, and it would also be ideal to use a treatment method that improves the likelihood of PSP resolution while reducing the risk of treatment-related adverse events. To our knowledge, no systematic review has compared PSP recurrence between conservative management and chest tube drainage as the initial treatment of PSP. In this systematic review and meta-analysis, we demonstrated that patients who received conservative management showed a comparable rate of PSP recurrence (pooled RR, 0.98; 95% CI, 0.75–1.28) and PSP resolution (pooled RR, 1.01; 95% CI, 0.9–1.15) to those who underwent chest tube drainage, but with fewer treatment-related adverse events (pooled RR, 0.22; 95% CI, 0.08–0.59).

Although asymptomatic patients with small PSP can be managed conservatively, there has been substantial heterogeneity in the management of moderate to large PSP [1]. Regarding cases of moderate to large PSP, the ACCP guideline recommends an invasive intervention with a small-bore catheter or chest tube to expand the lung [2], while the BTS suggests conservative management with observation, with chest drainage reserved for only physiologically significant patients [1]. Indeed, chest tube drainage has traditionally been considered the definitive treatment for PSP and has been the main procedure used for PSP treatment [1,2], but previous studies have also reported evidence supporting conservative management [15,22,23,24]. The choice of a treatment option for PSP is clinically relevant since chest tube drainage can cause treatment-related complications, prolong hospitalization, and put a financial burden on patients [11]. In this regard, our results support the concept that conservative management can be used as the initial treatment option for PSP because it yields a comparable recurrence and resolution rate of PSP, with fewer treatment-related adverse events than those occurring after chest tube drainage. However, our results should not be interpreted as providing evidence that conservative management can be applied to clinically significant symptomatic patients or physiologically unstable patients. Those patients should immediately undergo active interventions including chest tube insertion to manage their symptoms and physiological instability [1,2].

According to a prior systematic review dealing with the recurrence of PSP, interventional procedures did not show a significant reduction in the recurrence rate compared to conservative management (28.5% in the interventional procedure group vs. 21.9% in the conservative management group; *p* = 0.353) [3]. However, the interventional procedure group of the prior study included both needle aspiration and thoracostomy [3], but the ACCP guideline acknowledges that needle aspiration is not appropriate for PSP management in any clinical circumstances [2]. Therefore, a study comparing conservative management to only chest tube drainage, which is the definitive treatment option for PSP, is needed [1,2]. In this context, our results could provide evidence that conservative management is comparable to chest tube drainage in terms of recurrence after treatment, which implies that conservative management can be an alternative to chest tube drainage in hemodynamically stable PSP patients.

Although this meta-analysis revealed no significant difference in PSP recurrence between the conservative management and chest tube drainage groups, our results still have limitations in that there were only two studies in which the conservative management group and the tube drainage group were equivalently allocated [11,16]. In the other six studies, the patients were not equivalently allocated according to their PSP amount, and patients with small pneumothorax tended to be managed conservatively [6,9,15,17,18,19]. In the analysis of PSP recurrence, heterogeneity across the eight studies was 57%, and substantial heterogeneity remained even after adjusting for potential influencing factors such as study design, allocation of the PSP amount in each management group, and assessment time for recurrence. We must admit that it was very difficult to address every factor leading to this heterogeneity because only one study was an RCT [11]. For the analysis of PSP resolution, only one study equally assigned PSP cases into the two different management groups regardless of PSP amount [11]. The other two studies had a tendency for small PSP patients to undergo conservative management, and for patients with moderate or large PSP to receive chest tube drainage [19,20]. This may reflect the tendency in clinical practice for patients with moderate to large PSP to undergo chest tube drainage and have the accumulated air in the pleural cavity removed without any delay. To overcome this limitation, further studies equivalently allocating PSP cases into conservative management and chest tube drainage groups should be performed.

There are several other limitations of this study. First, there was only one RCT in this meta-analysis and this might cause a risk of bias in that the data from RCTs and non-RCTs were treated equivalently. The readers should pay attention to this limitation. Second, there was significant heterogeneity in the methods used to measure the amount of PSP between studies, and we could not take this issue into account. Third, only a small number of studies analysed PSP resolution and adverse events. Therefore, the statistical power of these analyses might not be sufficient to draw firm conclusions. Fourth, this review may not fully reflect advanced modern interventional techniques and medical treatments, since we included all studies regardless of their publication year.

## 5. Conclusions

In conclusion, as the initial treatment for PSP, conservative management is comparable to chest tube drainage in terms of PSP recurrence and resolution after treatment, with fewer adverse events during treatment.

## Figures and Tables

**Table 1:** Characteristics of studies included in the meta-analysis.

Authors	Years	Study Design	Allocation of the PSP Amount in Each Management Group	Assessment Time of PSP Recurrence	Inclusion of Outcome	Recurrence (Conservative Management)	Recurrence (Chest Tube Drainage)	Resolution (Conservative Management	Resolution (Chest Tube Drainage)	Adverse Event (Conservative Management	Adverse Event (Chest Tube Drainage)
Alfageme I et al.	1994	Prospective cohort	Non-equal	Within 12 months	Recurrence	7/21	11/34	N/A	N/A	N/A	N/A
Kelly AM et al.	2008	Retrospective cohort	Non-equal	N/A	Resolution	N/A	N/A	72/91	47/64	N/A	N/A
Ganesalingam R et al.	2010	Retrospective cohort	Equal	After 12 months	Recurrence	4/5	50/95	N/A	N/A	N/A	N/A
Kuan WS et al.	2011	Retrospective cohort	Non-equal	N/A	Resolution, adverse events	N/A	N/A	15/22	21/40	0/22	5/40
Chew R et al.	2014	Retrospective cohort	Equal	After 12 months	Recurrence, adverse events	6/53	6/58	N/A	N/A	0/53	16/58
Kim et al.	2014	Retrospective cohort	Non-equal	After 12 months	Recurrence	2/16	7/39	N/A	N/A	N/A	N/A
Olsen WH et al.	2016	Prospective cohort	Non-equal	Within 12 months	Recurrence	16/23	126/234	N/A	N/A	N/A	N/A
Huang YH et al.	2017	Retrospective cohort	Non-equal	After 12 months	Recurrence	1139/5277	1462/5679	N/A	N/A	N/A	N/A
Brown SGA et al.	2020	Randomized controlled trial	Equal	Within 12 months	Recurrence, resolution, adverse events	14/159	25/149	118/125	129/131	13/162	41/154
Balta C et al.	2020	Retrospective cohort	Non-equal	Within 12 months	Recurrence	0/24	2/56	N/A	N/A	N/A	N/A

PSP, primary spontaneous pneumothorax; N/A, not available.

## Supplementary Materials

The following are available online at https://www.mdpi.com/2077-0383/9/11/3456/s1, Table S1: Meta-regression comparing conservative management and chest tube drainage for primary spontaneous pneumothorax (PSP), Figure S1: Subgroup analysis comparing conservative management and chest tube drainage in terms of primary spontaneous pneumothorax recurrence (PSP). (A) Study design, (B) allocation of the PSP size, and (C) assessment time, Figure S2: Funnel plot of included studies evaluating recurrence of primary spontaneous pneumothorax, Figure S3: Subgroup analysis comparing conservative management and chest tube drainage in terms of primary spontaneous pneumothorax resolution (PSP). (A) Study design, (B) allocation of the PSP size, Figure S4: Subgroup analysis comparing conservative management and chest tube drainage in terms of adverse events during treatment. (A) Study design, (B) allocation of the primary spontaneous pneumothorax size.
