# Supplementary material for: Chest Tube Drainage Versus Conservative Management as the Initial Treatment of Primary Spontaneous Pneumothorax: A Systematic Review and Meta-Analysis
Source: J Clin Med. 2020 Oct 27;9(11):3456. doi: 10.3390/jcm9113456 (PMC7693596; doi:10.3390/jcm9113456)
Supplement: Supplementary file 1 [file jcm-09-03456-s001.pdf]

Table S1. Meta-regression comparing conservative management and chest tube drainage for primary spontaneous pneumothorax (PSP).

Outcome	Factor	Regression coefficient	P-value
Recurrence of PSP	Study design	0.076	0.816
	Allocation of PSP size	0.484	0.191
	Assessment time	0.076	0.816
Resolution of PSP	Study design	0.147	0.094
	Allocation of PSP size	0.147	0.094
Adverse events	Study design	−1.426	0.178
	Allocation of PSP size	0.055	0.98

PSP: primary spontaneous pneumothorax. Factors: study design (prospective vs. retrospective), allocation of the PSP size in each management group (equally allocated study vs. non-equally allocated study), and assessment time for recurrence (within 12 months vs. after 12 months).

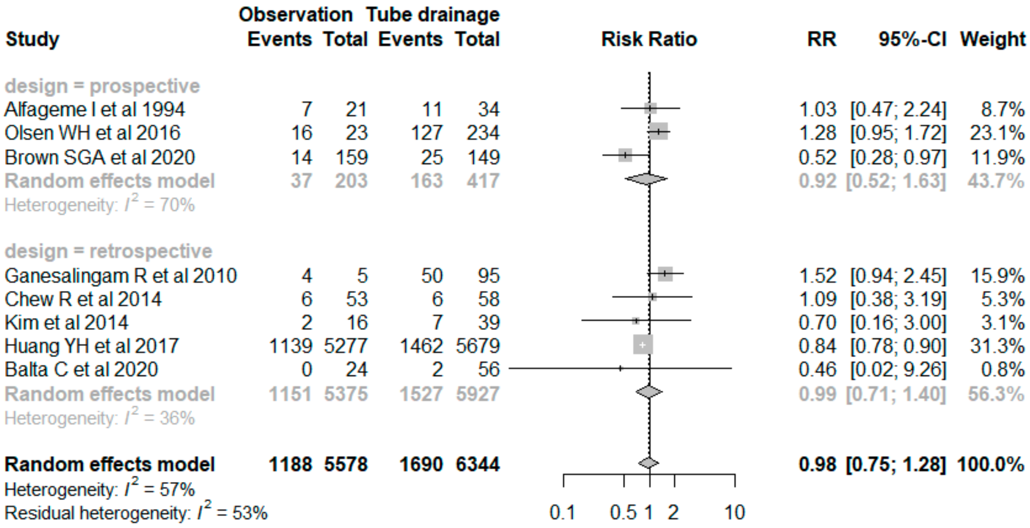
Study
Observation Events Total
Tube drainage Events Total
Risk Ratio
RR 95%-CI Weight
design = prospective
Alfageme I et al 1994 7 21 11 34 1.03 [0.47; 2.24] 8.7%
Olsen WH et al 2016 16 23 127 234 1.28 [0.95; 1.72] 23.1%
Brown SGA et al 2020 14 159 25 149 0.52 [0.28; 0.97] 11.9%
Random effects model 37 203 163 417 0.92 [0.52; 1.63] 43.7%
Heterogeneity: I^2 = 70%
design = retrospective
Ganesalingam R et al 2010 4 5 50 95 1.52 [0.94; 2.45] 15.9%
Chew R et al 2014 6 53 6 58 1.09 [0.38; 3.19] 5.3%
Kim et al 2014 2 16 7 39 0.70 [0.16; 3.00] 3.1%
Huang YH et al 2017 1139 5277 1462 5679 0.84 [0.78; 0.90] 31.3%
Balta C et al 2020 0 24 2 56 0.46 [0.02; 9.26] 0.8%
Random effects model 1151 5375 1527 5927 0.99 [0.71; 1.40] 56.3%
Heterogeneity: I^2 = 36%
Random effects model 1188 5578 1690 6344 0.98 [0.75; 1.28] 100.0%
Heterogeneity: I^2 = 57%
Residual heterogeneity: I^2 = 53%
0.1 0.5 1 2 10

(**A**)

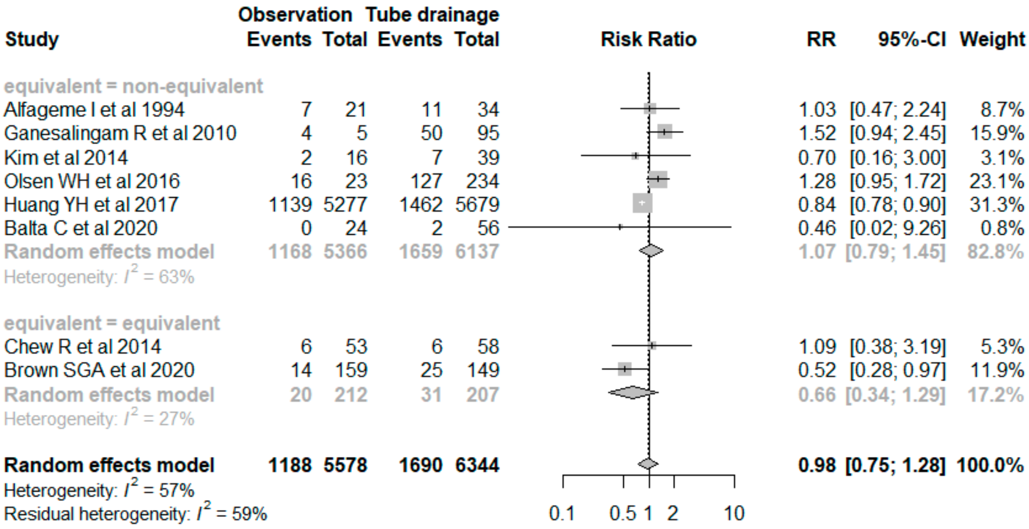
Study
Observation Events Total
Tube drainage Events Total
Risk Ratio
RR 95%-CI Weight
equivalent = non-equivalent
Alfageme I et al 1994 7 21 11 34 1.03 [0.47; 2.24] 8.7%
Ganesalingam R et al 2010 4 5 50 95 1.52 [0.94; 2.45] 15.9%
Kim et al 2014 2 16 7 39 0.70 [0.16; 3.00] 3.1%
Olsen WH et al 2016 16 23 127 234 1.28 [0.95; 1.72] 23.1%
Huang YH et al 2017 1139 5277 1462 5679 0.84 [0.78; 0.90] 31.3%
Balta C et al 2020 0 24 2 56 0.46 [0.02; 9.26] 0.8%
Random effects model 1168 5366 1659 6137 1.07 [0.79; 1.45] 82.8%
Heterogeneity: I^2 = 63%
equivalent = equivalent
Chew R et al 2014 6 53 6 58 1.09 [0.38; 3.19] 5.3%
Brown SGA et al 2020 14 159 25 149 0.52 [0.28; 0.97] 11.9%
Random effects model 20 212 31 207 0.66 [0.34; 1.29] 17.2%
Heterogeneity: I^2 = 27%
Random effects model 1188 5578 1690 6344 0.98 [0.75; 1.28] 100.0%
Heterogeneity: I^2 = 57%
Residual heterogeneity: I^2 = 59%
0.1 0.5 1 2 10

(**B**)

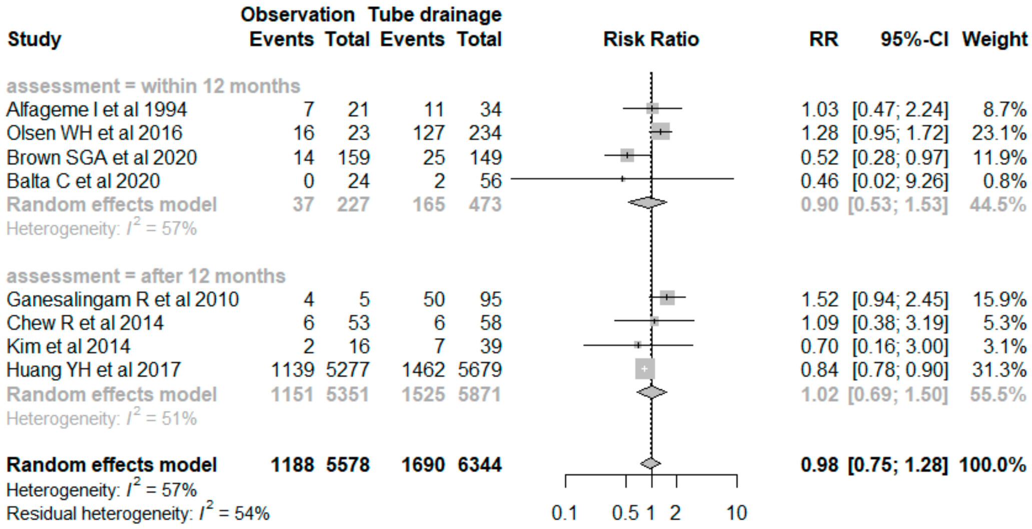

(**C**)

Figure S1. Subgroup analysis comparing conservative management and chest tube drainage in terms of primary spontaneous pneumothorax recurrence (PSP). (**A**) Study design, (**B**) allocation of the PSP size, and (**C**) assessment time.

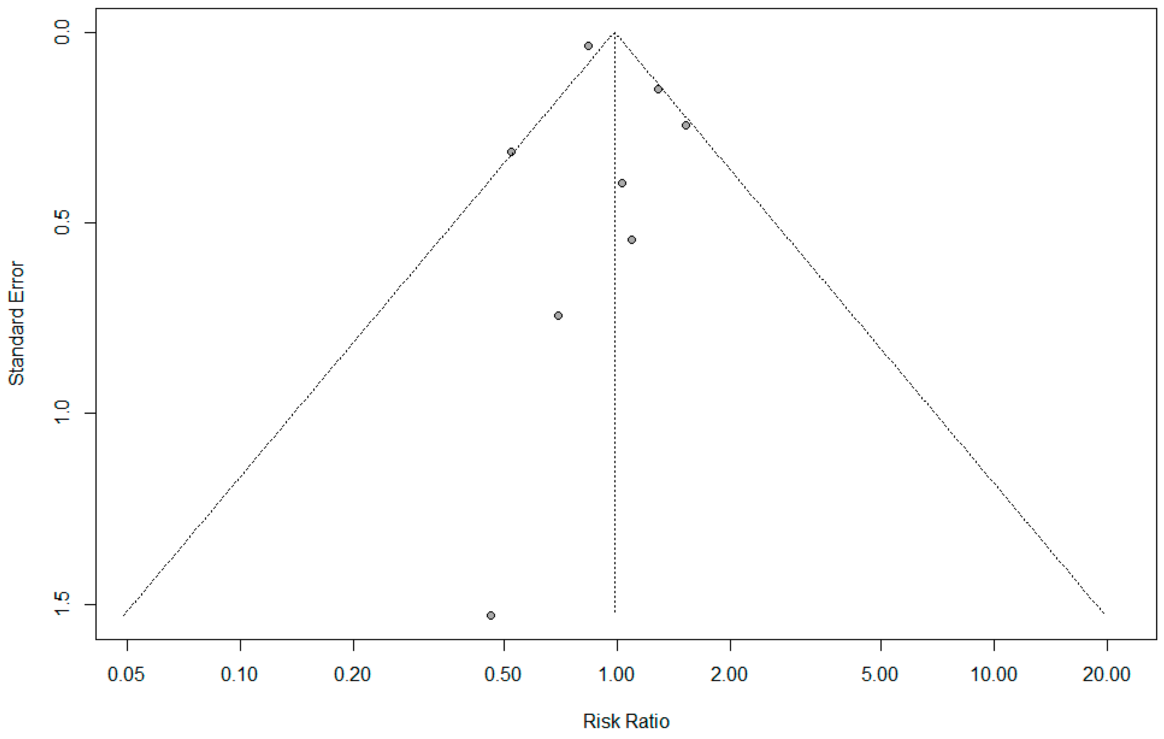

Figure S2. Funnel plot of included studies evaluating recurrence of primary spontaneous pneumothorax.

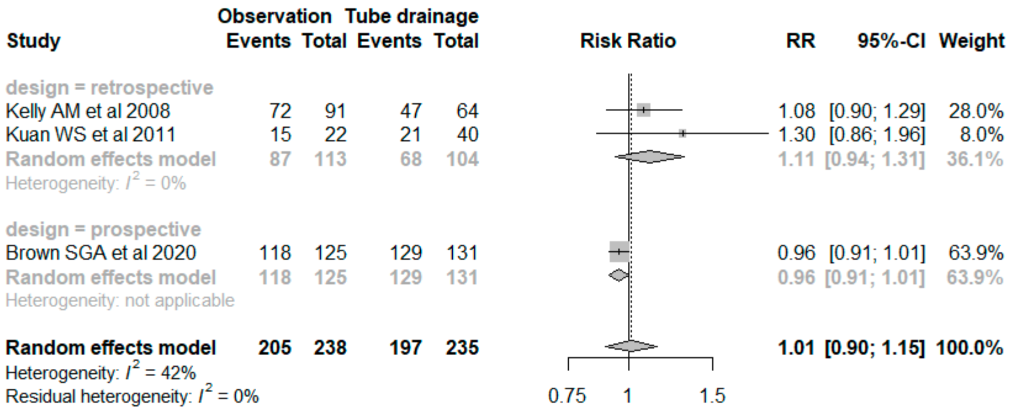

(A)

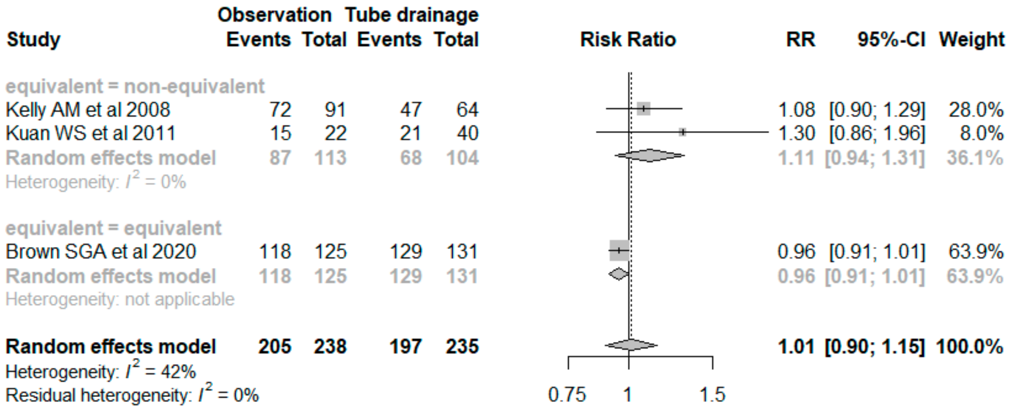

(B)

Figure S3. Subgroup analysis comparing conservative management and chest tube drainage in terms of primary spontaneous pneumothorax resolution (PSP). (**A**) Study design, (**B**) allocation of the PSP size.

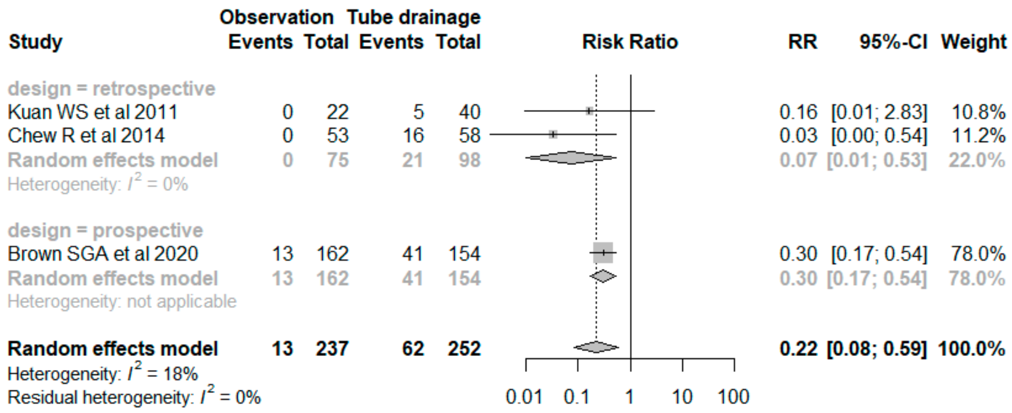

(A)

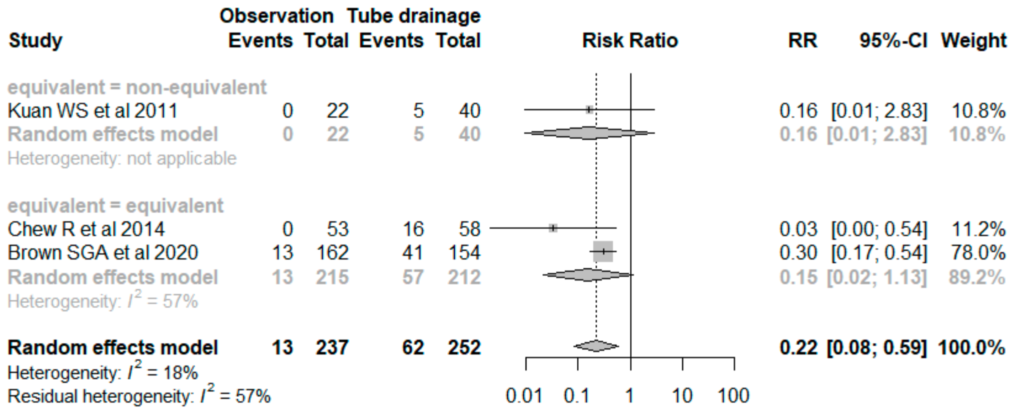

(B)

Figure S4. Subgroup analysis comparing conservative management and chest tube drainage in terms of adverse events during treatment. (**A**) Study design, (**B**) allocation of the primary spontaneous pneumothorax size.
